# Relationship between the Presence of the *ApoE ε*4 Allele and EEG Complexity along the Alzheimer’s Disease Continuum

**DOI:** 10.3390/s20143849

**Published:** 2020-07-10

**Authors:** Víctor Gutiérrez-de Pablo, Carlos Gómez, Jesús Poza, Aarón Maturana-Candelas, Sandra Martins, Iva Gomes, Alexandra M. Lopes, Nádia Pinto, Roberto Hornero

**Affiliations:** 1 Biomedical Engineering Group, E.T.S.I. de Telecomunicación, Universidad de Valladolid, 47011 Valladolid, Spain; victor.gutierrez@gib.tel.uva.es (V.G.-d.P.); jesus.poza@tel.uva.es (J.P.); aaron.maturana@gib.tel.uva.es (A.M.-C.); robhor@tel.uva.es (R.H.); 2 Centro de Investigación Biomédica en Red en Bioingeniería, Biomateriales y Nanomedicina, (CIBER-BBN), 28029 Madrid, Spain; 3 Instituto de Investigación en Matemáticas (IMUVA), Universidad de Valladolid, 47011 Valladolid, Spain; 4 Institute of Molecular Pathology and Immunology of the University of Porto (IPATIMUP), 4200-135 Porto, Portugal; smartins@ipatimup.pt (S.M.); igomes@ipatimup.pt (I.G.); alopes@ipatimup.pt (A.M.L.); npinto@ipatimup.pt (N.P.); 5 Institute of Research and Innovation in Health (i3S), University of Porto, 4200-135 Porto, Portugal; 6 Center of Mathematics of the University of Porto (CMUP), 4169-007 Porto, Portugal

**Keywords:** Alzheimer’s disease (AD), apolipoprotein E (ApoE), electroencephalography (EEG), Lempel-Ziv complexity (LZC), mild cognitive impairment (MCI)

## Abstract

Alzheimer’s disease (AD) is the most prevalent cause of dementia, being considered a major health problem, especially in developed countries. Late-onset AD is the most common form of the disease, with symptoms appearing after 65 years old. Genetic determinants of AD risk are vastly unknown, though, ε4 allele of the *ApoE* gene has been reported as the strongest genetic risk factor for AD. The objective of this study was to analyze the relationship between brain complexity and the presence of *ApoE*ε4 alleles along the AD continuum. For this purpose, resting-state electroencephalography (EEG) activity was analyzed by computing Lempel-Ziv complexity (LZC) from 46 healthy control subjects, 49 mild cognitive impairment subjects, 45 mild AD patients, 44 moderate AD patients and 33 severe AD patients, subdivided by *ApoE* status. Subjects with one or more *ApoE*ε4 alleles were included in the carriers subgroups, whereas the *ApoE*ε4 non-carriers subgroups were formed by subjects without any ε4 allele. Our results showed that AD continuum is characterized by a progressive complexity loss. No differences were observed between AD *ApoE*ε4 carriers and non-carriers. However, brain activity from healthy subjects with *ApoE*ε4 allele (carriers subgroup) is more complex than from non-carriers, mainly in left temporal, frontal and posterior regions (*p*-values < 0.05, FDR-corrected Mann–Whitney *U*-test). These results suggest that the presence of *ApoE*ε4 allele could modify the EEG complexity patterns in different brain regions, as the temporal lobes. These alterations might be related to anatomical changes associated to neurodegeneration, increasing the risk of suffering dementia due to AD before its clinical onset. This interesting finding might help to advance in the development of new tools for early AD diagnosis.

## 1. Introduction

Dementia due to Alzheimer’s disease (AD) is characterized by progressive decline in several areas of cognition, including memory, language and executive functions. The percentage of people with AD increases greatly with age, ranging from 3% in people between 65 and 74 years to 32% in people over 85 years [1]. In 2018, over 50 million population dealt with some kind of dementia, and an increase to 152 million people is estimated by 2050 [2]. Accumulating evidence suggests that AD starts developing in brain 20 years or more before first symptoms, with small, but detectable, alterations [1]. Three main stages can be distinguished: mild AD (AD${}_{MIL}$), moderate AD (AD${}_{MOD}$), and severe AD (AD${}_{SEV}$). AD${}_{MIL}$ is characterized by symptomatic issues as memory loss, language problems, mood changes and decreased judgement arise. In the middle stage, AD${}_{MOD}$, basic functions are affected, hindering patient performance. In the most severe phase, AD${}_{SEV}$ patients are totally dependent on their caregivers and have their verbal and psychomotor skills lost [3]. An intermediate state between normal aging and AD has been described as mild cognitive impairment (MCI) [4,5]. This term is used to refer to individuals who show slight memory impairments and declined thinking skills, but do not meet dementia due to AD diagnostic criteria [4]. MCI has been accepted as a prodromal stage of the disease, since 15–18% of people over 60 years tend to develop MCI, with 8–15% of them evolving to dementia every year [4].

As for the molecular pathogenesis, AD presents several features, such as: neuritic plaques, composed of aggregated amyloid beta protein (Aβ); and neurofibrillary tangles, formed by hyperfosforilated tau protein [5]. In 2018, the National Institute of Aging and Alzheimer’s Association (NIA-AA) defined a new method to unify the diagnosis of preclinical dementia, MCI and dementia due to AD in different stages, using the presence of Aβ, tau protein and neuronal injuries to classify patients into different profiles [6]. Late-onset AD is the most common form of the disease, approximately 20 times more prevalent than early onset AD [7]. It appears in sporadic cases after 65 years old and is a complex disease, with a large number of underlying genetic factors. Tens of genetic variants have been recently associated with the disease through genome wide analyses in tens of thousands of subjects [8,9]. Among them, the largest and most consistent effect has been observed in *apolipoprotein E* (*ApoE*), with the ε4 allele the strongest genetic risk factor for developing AD [10,11]. The *ApoE* protein is composed of 299 amino acids and variation in two polymorphic sites results in three isoforms: *ApoE2* (Cys112, Cys158), *ApoE3* (Cys112, Arg158), and *ApoE4* (Arg112, Arg158), encoded by the ε2, ε3 and ε4 alleles of the *ApoE* gene, respectively [12]. Individuals with one ε4 allele are roughly 4 times more likely to develop AD, and homozygous ε4/ε4 have about 15-times-increased risk, when compared with individuals carrying the most common genotype ε3/ε3 [13].

Different neuroimaging techniques have been used to study these biological changes in the AD patients’ brain, such as functional magnetic resonance imaging (fMRI) and positron emission tomography (PET) [5]. fMRI is a sensitive, non-invasive technique, which detects the flow and the oxigenation level of the blood; it is used to assess AD-associated cognitive task-related changes in brain activity [14,15]. On the other hand, PET quantifies the deposition of Aβ plaques using amyloid agents or radiotracers [14]. Both fMRI and PET have limitations, notwithstanding, due to high costs and the need of intravenously injection of radiotracers for PET acquisition [16,17]. For these reasons, the study of alternative low-cost non-invasive methods is of upmost importance for an early diagnosis of AD. Electroencephalography (EEG) is a widely-used low-cost technique that measures brain electrical activity [15]. It is characterized by a high temporal resolution, which is useful to record the transient and highly dynamic nature of brain oscillations. EEG analyses have provided novel insights on how dementia due to AD affects neural activity [18]. In this regard, previous EEG studies showed that MCI and AD patients exhibited higher power in low-frequency bands, and lower in high-frequency bands, than healthy controls (HC), suggesting that dementia due to AD produces a slowing of neural oscillations [19,20]. In addition, EEG coherence has been used to detect alterations in brain regional connectivity; specifically, a decrease of alpha coherence in temporal, parietal and occipital regions and an increase of delta coherence in frontal and posterior regions for AD patients [21]. Moreover, non-linear analyses also identified changes in brain patterns through the analysis of entropy metrics and complexity measures, associating AD with a decrease irregularity and complexity of brain activity [22,23,24,25,26,27,28,29].

Several studies have studied the relationship between EEG measurements and *ApoE* ε4 allele [30,31,32,33]. Ponomareva et al. [30] analyzed resting EEG signals in AD patients, unaffected first degree relatives of theirs, and unrelated control subjects, stratifying them by *ApoE* genotype. Resting EEG parameters of AD relatives and age-matched healthy controls showed to be indistinguishable. Among AD patients, ε4 carriers showed a higher decrease of alpha relative power than ε4 non-carriers. Interestingly, under hyperventilation, the presence of ε4 allele in AD relatives was associated to the manifestation of synchronous high-amplitude delta and theta activity and sharp waves, a decrease of relative power in alpha and an increase in delta and theta frequency bands. In other study, Canuet et al. [31] assessed correlations between spatial patterns and *ApoE* genotype on resting-state oscillations and functional connectivity in AD patients. AD ε4 carriers showed reduced alpha activity in the left inferior parietal and temporo-occipital cortex compared to non-carriers. In addition, a decreased alpha 2 (10–13 Hz) connectivity pattern in AD was reported, involving the left temporal and bilateral parietal cortex. Moreover, several brain regions exhibited an increased lagged phase synchronization in low frequencies, especially in the theta band, across and within hemispheres, where temporal lobe connections were mostly affected. Kramer et al. [32] evaluated the relation between the *ApoE*ε4 genotype and functional connectivity in patients with AD and subjects with subjective complaints. *ApoE*ε4 carriers showed higher synchronization likelihood values in lower and upper alpha bands (8–10 Hz and 10–12 Hz, respectively) in both subjective complaints and AD patients. Moreover, in upper alpha and beta bands AD patients presented lower synchronization values than subjects with subjective complaints, independently from *ApoE* status. On the other hand, Zappasodi et al. [33] studied the correlation between *ApoE*ε4 genotype effect and free copper toxicosis. Higher levels of copper were found in AD patients than in HC subjects and correlated positively with parieto-temporal delta and negatively with parieto-temporal alpha 1 activity (8–10.5 Hz). Furthermore, peroxide levels correlated with higher temporal delta activity in AD patients [33]. In summary, previous studies analyzed the relationship between *ApoE* genotype and brain activity by means of spectral measures [30,33] and functional connectivity metrics [31,32]. However, the relationship between the non-linear properties of the EEG and *ApoE* gene has not been explored yet. As non-linearity is an inherent property of the brain [34], non-linear techniques (i.e., entropy methods and complexity estimators) would give a better understanding on how *ApoE* genotype affects to the non-linear behavior of the neural dynamics [35]. Entropy methods, such as approximate entropy, sample entropy or permutation entropy, have been previously used to quantify the EEG irregularity loss in AD [22,25]. However, these methods cannot fully express the physiological dynamics of more complex processes of the brain [36]. On the other hand, complexity estimators have been successfully applied to characterize the brain alterations in AD [26,27]. Therefore, it seems reasonable to apply non-linear parameters and, among them, complexity methods, to quantify the effect of *ApoE* gene on EEG activity. Although several complexity methods can be employed for this purpose (i.e., correlation dimension, multiscale entropy, Higuchi’s fractal dimension, etc.), in this study we will apply Lempel–Ziv complexity (LZC) due to its advantages over other complexity estimators: (i) LZC algorithm is very simple to program and compute; (ii) a normalized complexity estimation of the time series can be obtained; (iii) LZC does not require long data segments to be calculated; (iv) for LZC estimation, no parameters need to be specified; and (v) LZC has proved its usefulness to detect complexity changes in AD patients’ neural signals [37,38,39,40,41].

Besides the relationship between *ApoE* genotype and the brain electrical activity, cognitive alterations have been previously found in *ApoE* ε4 carriers using fMRI, reporting decreased performance and faster cognitive decline compared to non-carriers [42]. Particularly, several studies on HC subjects have shown that ε4 carriers present abnormal cognitive functions and connectivity patterns while performing the same tasks as non-carriers, not only in old age, but since childhood [43,44,45,46,47,48]. Analyzing these studies, a common point can be found: the medial temporal lobes (MTLs). There is abundant evidence that MTL is the earliest site of AD-associated pathology, since the neurofibrillary tangles characteristic of AD are initially found in there, being later spread dorsolaterally over the cortex [49,50]. Furthermore, there is evidence of greater susceptibility of the left hemisphere to degenerative brain disease due to predominant hypometabolism in this side of the brain [51], which means that these cognitive alterations affect the brain areas differently. These two premises may help to evaluate how *ApoE*ε4 effect is associated with EEG complexity changes in the left temporal lobe.

The aim of this research was to evaluate the effect of the presence of *ApoE* ε4 alleles on EEG along the AD continuum. *ApoE* risk allele effect has been study using fMRI [43,44,45,46,47,48] or spectral analysis methods [19,30,32,33]; however, the present research goes a step further by exploring the association between *ApoE*ε4 allele and non-linear patterns of neural activity across AD progression. Specifically, in the current study we address the following research questions: (i) does *ApoE*ε4 status alter complexity patterns of neural activity? (ii) what are the spatial patterns associated to the alterations in the complexity of EEG that can be linked to this genotype? and (iii) is it possible to establish a relationship between the alterations in complexity EEG patterns and *ApoE* risk allele in the left temporal lobe along the AD continuum?

## 2. Materials and Methods

### 2.1. Subjects

A total of 217 subjects participated in this study: 46 HC, 49 individuals with MCI, and AD patients in different stages of the disease (45 AD${}_{MIL}$, 44 AD${}_{MOD}$, and 33 AD${}_{SEV}$). MCI and dementia due to AD patients were diagnosed in accordance with NIA-AA criteria [52,53], being AD stages established through the Mini-Mental State Examination (MMSE) test [54]. HC were individuals older than 68, with no signs of dementia and no history of neurological or major psychiatric disorders, and MMSE scores higher than 27. Exclusion criteria include history of active or treated neoplasia, history of recent surgery or hypercatabolic states, severe alcoholism or indications of vascular disease in clinical history, as for previous studies [25,55]. All the subjects were unrelated among them and were residents of the North of Portugal or the autonomous region of Castile and Leon, Spain. All subjects, legal representatives, family and/or caregivers gave their written consent to join the study, in accordance with the recommendations of the Code of Ethics of the World Medical Association (Declaration of Helsinki). The project was approved by the Ethics Committee of the University of Porto, Portugal (Report n.${}^{{}^{\circ}}$ 38/CEUP/2018).

Each of the five groups under study (HC, MCI, AD${}_{MIL}$, AD${}_{MOD}$, and AD${}_{SEV}$) were divided into two subgroups according to the *ApoE*ε4 status: carriers subgroups included subjects with one or more ε4 alleles, whereas non-carriers subgroups consisted of subjects without any ε4 allele [30]. Table 1 summarizes relevant socio-demographic and MMSE data of the participants. No statistically significant differences between *ApoE*ε4 carriers and non-carriers subgroups were found neither for age (*p*-values > 0.05, Mann–Whitney U-test), nor gender (*p*-values > 0.05, Chi-square test), nor MMSE (*p*-values > 0.05, Mann–Whitney U-test).

### 2.2. ApoE Genotyping

Genome analysis was carried out by collecting saliva from all subjects with the Oragene DNA (OG-500) self-collection kit (DNA Genotek, Ottawa, ON, Canada); alternatively, cotton-steriled buccal swabs were used for patients in more advanced stages of the disease. DNA from the saliva samples, collected with the Oragene kit, was extracted using the prepIT DNA extraction kit (DNA Genotek, Ottawa, ON, Canada), and from the buccal swabs using Citogene extraction kit (Citomed, Odivelas, Portugal) both following the manufacturer’s instructions. *ApoE* alleles were determined by sequencing of the variants, SNPs rs74389 and rs7412 using a traditional Sanger sequencing protocol.

### 2.3. EEG Recording

For each participant, five minutes of resting-state EEG activity were registered using a 19-channel Nihon Kohden Neurofax JE-921A EEG System at a sampling rate of 500 Hz. Electrode layout followed the specifications of the International 10-20 System, including the following positions: Fp1, Fp2, F3, F4, C3, C4, P3, P4, O1, O2, F7, F8, T3, T4, T5, T6, Fz, Cz and Pz. EEG recordings were carried out using a common average reference. Subjects were asked to remain eye-closed and awake in a noise-free enviroment to minimize artifact presence. If signs of somnolence were found, subjects were asked to stay awake during the acquisition process.

For each five-minute EEG recording, the next pre-processing steps were followed [25,55]: (i) mean removal; (ii) electrical power line attenuation with 50 Hz notch filter; (iii) bandpass finite impulse response (FIR) filtering with Hamming window from 0.4 to 70 Hz; (iv) independent component analysis (ICA) to eliminate artifact components related to myographic, cardiographic and oculographic noise; (v) segmentation in 5 s epochs; and (vi) visual rejection of artifacted epochs.

### 2.4. Lempel-Ziv Complexity

LZC is a simple-to-compute non-parametric measure of complexity for one-dimensional sequences of finite length [37,56]. It is related to the number of different substrings and the rate of recurrence along the given sequence, with larger values according to more complex data [26].

Before computing the complexity measure c(n), the temporal signal must be converted into a binary sequence P=s(1)‚s(2)‚…‚s(n) comparing the signal with a threshold $T_{d}$, calculated as the median value due to its robustness to outliers [57,58]. Then, s(i) is defined by [57]:

(1) $$s\left(i\right)=\left\{\begin{array}{cc} 0 & if\;x\left(i\right)<T_{d}\\ 1 & if\;x\left(i\right)\geq T_{d} \end{array}\right.‚$$

where x(i) is the *i*-th sample of the original temporal signal.

The discrete sequence *P* is checked from left to right and the complexity counter c(n) is increased by one every time a new subsequence of consecutive elements is found. This measure can be calculated by the following method [37,56,57,58]:

i. Let *S* and *Q* denote different subsequences of *P* and SQ be the concatenation of *S* and *Q*. On the other hand, sequence SQπ is derived from SQ after the deletion of its last element (π means the operation to eliminate the last element in the sequence). Let v(SQπ) denote the vocabulary of all different subsequences of SQπ. At the beginning, c(n)=1, S=s(1), Q=s(2), consequently, SQπ=S(1).
ii. In general, S=s(1)‚s(2)‚…‚s(m), Q=s(m+1), then SQπ=s(1)‚s(2)‚…‚s(m); if *Q* belongs to v(SQπ), *Q* is a subsequence of SQπ, not a new sequence.
iii. Renew *Q* as Q=s(m+1), s(m+2) and check if *Q* belongs to v(SQπ) or not.
iv. Repeat the same steps until *Q* does not belong to v(SQπ). Then *Q* equal to s(m+1)‚s(m+2)‚…‚s(m+i) is not a subsequence of SQπ=s(1)‚S(2)‚…‚s(r+i−1), so increase c(n) by one.
v. After that, renew S=s(1)‚s(2)‚…‚s(r+i) and Q=s(r+i+1).

These steps have to be repeated until *Q* is the last element of the sequence. Then, the number of different subsequences in *P* is c(n). To obtain a complexity measure totally independent of the sequence length, the parameter c(n) should be normalized. If the length of the original sequence is *n*, it has been proved in [56] that the upper limit of c(n), which is denoted as b(n), can be expresed by:

(2) $$b\left(n\right)\equiv \frac{n}{log_{2}\left(n\right)}‚$$

and c(n) can be normalized by b(n):

(3) $$C\left(n\right)=\frac{c(n)}{b(n)}‚$$

where C(n) is the normalized LZC.

### 2.5. Statistical Analysis

Initially, a descriptive analysis was carried out to assess normality and homocedasticity of data distribution. Lilliefors test was performed to evaluate the normality of the data, and Bartlett test was used to study the homogeneity of variances. As LZC results did not meet the parametric test conditions, a non-parametric test was used. For this reason, statistical differences between different groups and between *ApoE* ε4 carrier and non-carrier subgroups were assessed with Mann–Whitney *U*-tests. A false discovery rate (FDR) correction was applied to avoid falsely rejected hypotheses [59]. Signal processing and statistical analyses were carried out using Matlab (version R2018a, Mathworks, Natick, MA, USA).

## 3. Results

### 3.1. Global Analysis

**Figure 1:**
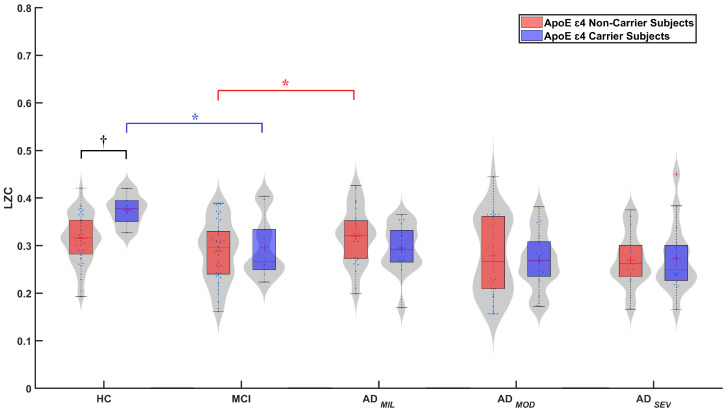
Global LZC distribution along AD continuum: in red, *ApoE* ε4 non-carrier subgroups; in blue, *ApoE*ε4 carrier subgroups. Statistically significant differences are indicated in the figure (*: differences along the AD continuum, *p*-value < 0.05; †: differences between ε4 carrier and non-carrier subjects, *p*-value < 0.05, Mann–Whitney *U*-test).

Grand-average LZC was computed for *ApoE* ε4 non-carrier and carrier subgroups. Figure 1 shows the distribution of LZC values for the ten subgroups (carrier and non-carrier subgroups for the five stages of AD continuum: HC, MCI, AD${}_{\mathrm{MIL}}$, AD${}_{\mathrm{MOD}}$, and AD${}_{\mathrm{SEV}}$). Mann–Whitney *U*-tests were computed to evaluate differences between groups along the AD continuum. In this way, statistically significant differences were found for the comparisons: MCI vs. AD${}_{MIL}$, for non-carriers subgroup (marked in Figure 1 with red brackets) (*p*-value = 0.0354, *U*-value = 1160, Mann–Whitney *U*-test); and HC vs. MCI, for the carriers subgroup (marked with blue brackets) (*p*-values = 0.0420, *U*-value = 70, Mann–Whitney *U*-test). For both subgroups, LZC decreases from normal aging to severe stages, suggesting a reduction of complexity with AD progression.

On the other hand, statistical analyses were carried out within each group and differences between non-carriers and carriers were observed in HC subjects (marked with black brackets) (*p*-value = 0.0065, *U*-value = 856, Mann–Whitney *U*-test). No differences were found between other subgroups.

### 3.2. Spatial Analysis

**Figure 2:**
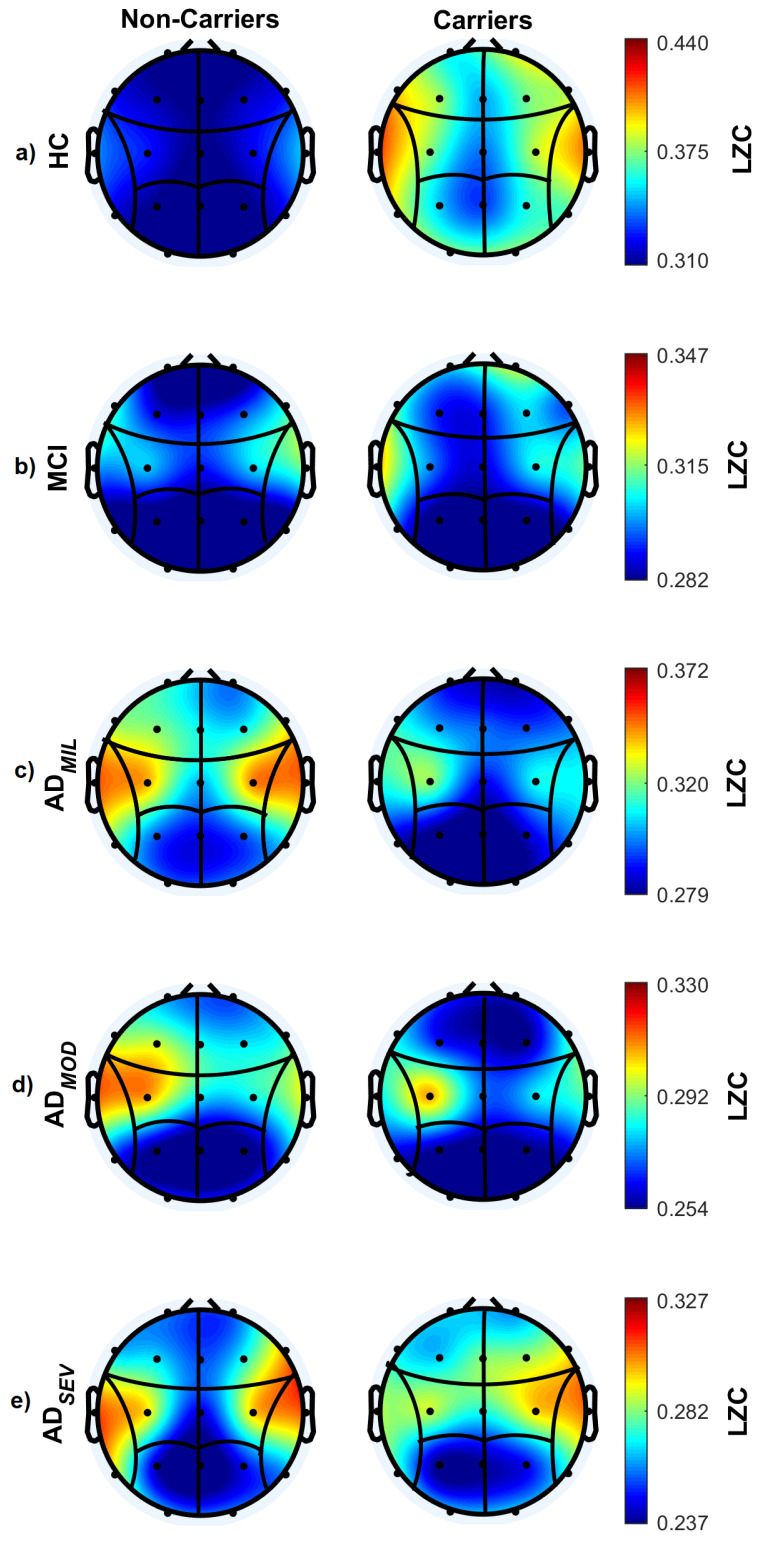
Lempel–Ziv complexity measured on each ROI for non-carrier and carrier subjects. Left diagrams represent average LZC values for *ApoE* ε4 non-carrier groups, whereas right diagrams depict average LZC values for *ApoE*ε4 carrier groups. (**a**) LZC values for HC subjects; (**b**) LZC values for MCI patients; (**c**) LZC values for AD${}_{MIL}$ patients; (**d**) LZC values for AD${}_{MOD}$ patients; (**e**) LZC values for AD${}_{SEV}$ patients.

In order to obtain local measurements of LZC, a spatial analysis was also performed. EEG channels were grouped into regions of interest (ROIs) [60,61]: left frontal (Fp1, F3, F7), right frontal (Fp2, F4, F8), left central (C3), right central (C4), left temporal (T3, T5), right temporal (T4, T6), left posterior (P3, O1), and right posterior (P4, O2). The results are presented in Figure 2. Compared to non-carrier subjects, HC carriers showed a higher complexity in all brain regions, particularly in temporal ROIs. In the case of the subgroup of MCI individuals, both carriers and non-carriers showed higher complexity levels in temporal lobes than the other subgroups, except HC subjects, with slightly high complexity values for carriers in right frontal ROI. Concerning AD patients, non-carriers showed higher LZC values than carriers, namely in temporal and central ROIs (AD${}_{MIL}$ patients), in left temporal ROIs (AD${}_{MOD}$ patients), and in temporal and central regions (AD${}_{SEV}$ patients).

A statistical analysis was carried out in each ROI to compare LZC values between carriers and non-carriers for each group. Statistically significant differences between HC subjects subgroups were found in left temporal, frontal and posterior regions (left temporal: *p*-value = 0.0242, *U*-value = 861; left frontal: *p*-value = 0.0242, *U*-value = 865; right frontal: *p*-value = 0.0242, *U*-value = 859; left posterior: *p*-value = 0.0242, *U*-value = 863; right posterior: *p*-value = 0.0242, *U*-value = 865; FDR-corrected Mann–Whitney *U*-tests).

## 4. Discussion

The aim of this study was to test for a relationship between EEG complexity changes due to AD and the dosage of the *ApoE* ε4 allele. For this purpose, resting-state EEG signals were analyzed in 46 control subjects, 49 individuals with MCI, 45 AD${}_{MIL}$ patients, 44 AD${}_{MOD}$ patients, and 33 AD${}_{SEV}$ patients, divided into two subgroups: *ApoE*ε4 carriers and non-carriers.

### 4.1. Loss of Complexity along AD Continuum

We first assessed whether the *ApoE* genotype affects to the complexity patterns of resting-state EEG activity, quantified using LZC. This complexity measure has been used in several studies on dementia due to AD, but none has yet analyzed its role to reflect alterations in neural patterns associated to the *ApoE* genotype. In this regard, our LZC results showed a complexity decrease in AD patients in comparison with HC subjects. Previous studies have also reported a loss of complexity in patients with dementia using different complexity measures, such as Higuchi’s fractal dimension, correlation dimension, Lyapunov exponents and neural complexity [26,27,28,29,37,62]. The neurophysiological implications of the EEG complexity reduction in AD patients is not clear, but it might be due to neuronal death, a general effect of neurotransmitter deficiency and/or connectivity loss of local neuronal networks [34].

In our study, differences found on EEG complexity between HC and MCI, and between MCI and AD${}_{MIL}$ groups showed a significant loss of complexity at the MCI development and as AD progresses, which are in line with previous studies [37,63]. Our findings suggest that the decrease on EEG complexity along the AD continuum occurs in both *ApoE*ε4 carriers and non-carriers subgroups. However, the alterations of non-linear neural patterns along AD continuum might be related with the *ApoE*ε4 status, since individuals who carry this allele are more likely to develop AD at younger ages than non-carriers [1]. In fact, this idea is supported by the statistical differences found between non-carrier and carrier HC subjects, which suggests that *ApoE*ε4 allele may be associated with cognitive disturbances even before the manifestation of AD symptoms. This condition has been described as preclinical AD stage, in which subjects do not show any impairment but display AD lesions on postmortem analysis, as abnormal levels of Aβ protein burden [6]. Thus, previous studies have analyzed whether Aβ deposition modifies both EEG and MEG patterns [64,65]. Their results indicate that brain activity is regulated according to Aβ levels, hypothesizing compensatory mechanisms to keep normal cognitive functions [64,65]. In line with these studies, our results suggest that *ApoE* risk allele effect could be associated with early abnormal depositions of Aβ protein, but also with other physiological alterations caused in preclinical AD. In order to check the effect of this gene, several previous studies analyzed the influence of ε4 status in neuroanatomic signatures of subjects in phases previous to AD development. Thereby, Duke Han et al. [48] explored HC older adults, finding that ε4 carriers subgroup displayed greater activation than non-carriers subgroup in multiple right hemisphere regions; whereas Shaw et al. [43] studied healthy children and adolescents, detecting differences in the cortical thickness of the left enthorinal region between ε4 carriers and non-carriers. Both studies found differences between ε4 carriers and non-carriers in stages previous to the development of AD, which is in line with our results.

### 4.2. Alterations in Spatial Patterns of Complexity Related to ApoE Genotype

To answer the second research question devoted to analyzing whether *ApoE* genotype modifies spatial patterns of EEG complexity, we performed a regional analysis. Previous research used different non-linear methods to assess the complexity decrease associated with AD progression and the brain areas involved [26,63,66]. Al-Nuaimi et al. [26] used three non-linear methods (Tsallis entropy, Higuchi Fractal Dimension, and LZC) to evaluate the complexity of each EEG channel and frequency band, obtaining lower complexity values for AD patients compared to HC in all frequency bands. Specifically, the most statistically significant differences were observed in temporal, parietal and occipital areas for LZC. In another EEG study, Zhu et al. [63] compared MCI and control subjects by means of LZC, observing that frontal and temporal complexity values decreased significantly when compared with other regions, which indicated that disturbances in neural activity were heterogeneously distributed across brain regions. This spatial-dependent patterns of alterations was also observed by Escudero et al. [67] that used the multiescale entropy to evaluate EEG complexity in AD progression; their results indicated that AD patients showed a lower complex activity in frontal, temporal, parietal and occipital regions when compared to controls. Although the altered areas differ from each research, that may be caused by different complexity methods and/or the heterogeneity of the databases, all these findings partially agree with our results. They reported higher complexity values in temporal, central and frontal regions in HC subjects, which are progressively reduced as AD advances. Interestingly, these results seem to suggest that, despite the fact that the EEG complexity decreases as the AD progresses, certain brain areas are relatively spared and still show higher complexity than other regions. This idea is supported by the decrease-of-complexity hyphotesis of Lipsitz and Goldberger, which explains that physiological diseases was linked to a generalized loss of complexity in the dynamics of healthy structures, which leads to functional loss and deficits [68].

We also analyzed the association between *ApoE* ε4 alleles and EEG complexity. Our results showed statistically significant differences between non-carrier and carrier HC subjects in left temporal, frontal and posterior regions. Several studies have suggested different hyphoteses for the presence of *ApoE*ε4 alleles on the brain physiology in healthy subjects [30,43,44,45,46,47,48]. Shaw et al. [43] used fMRI to study physiological changes in healthy children and adolescents; they observed that the left enthorinal region was significantly thinner in ε4 carriers than in non-carriers, which might be contributing to an increased risk of developing AD potentially identificable since childhood. Machulda et al. [47] examined the default mode network and the salience network taking into account the *ApoE*ε4 status in elderly HC. Carrier subjects showed reduced connectivity of the posterior default mode network, especially in left MTL, and an increased connectivity in the salience network. In another study, Duke Han et al. [48] investigated the association between the *ApoE* genotype and brain responses to verbal paired-associated learning in cognitively normal older adults. *ApoE*ε4 carrier group showed greater activation in right hemisphere areas than non-carrier subjects, suggesting that compensatory mechanisms related to a right hemisphere regions network might be involved. Bookheimer et al. [44] evaluated the effect of the *ApoE* genotype in healthy subjects performing memory tasks in comparison with resting periods, and observed that, in learning or recall periods, MRI signal intensity increased in left inferior frontal region, right prefrontal cortex, transverse temporal gyri, left posterior temporal region, and inferior parietal region. However, the intensity of activation in learning or recall periods was greater in *ApoE*ε4 carriers than in non-carriers. All these conclusions can be supported by our results, explaining differences between LZC values of ε4 non-carrier and carrier HC subjects based on neuroanatomical changes, which might indicate more susceptibility to neurodegeneration, revealing potential risk of possible dementia before its clinical onset.

### 4.3. Alterations in the Left Temporal Lobe Related to ApoE Genotype

**Figure 3:**
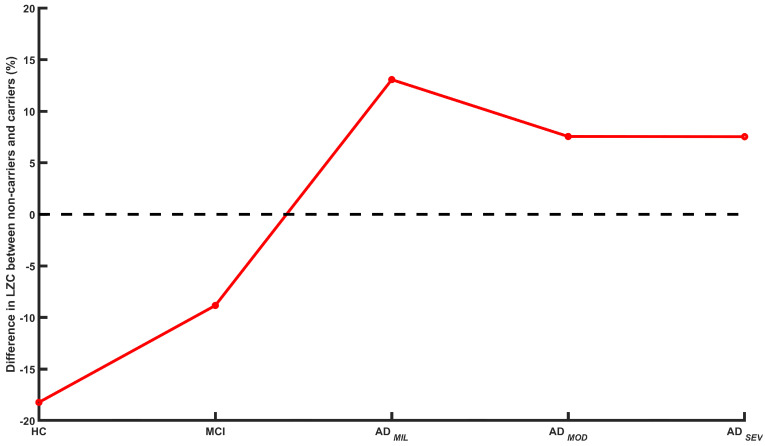
Average differences in the left temporal ROI between LZC values of *ApoE* ε4 non-carrier and carrier subjects along the AD continuum. Differences between LZC values are computed as: 100 × (non-carrier value − carrier value)/carrier value.

Finally, the third research question addressed the role of the left temporal lobe to establish a relationship between the EEG complexity patterns and *ApoE* ε4 allele. It is known that MTL structures are the earliest brain regions of AD-associated pathology [50]. For this reason, significant differences in the measurements observed in HC left temporal lobe may be a consequence of neuropathological alterations in these regions. As seen in Figure 3, a relationship between LZC in left temporal ROI and ε4 allele dosage can be established. LZC values in *ApoE*ε4 non-carriers are lower in this brain region than in carriers for HC group. Along the AD continuum, this difference (non-carriers minus carriers) is still negative in MCI stage; however, it becomes positive for the three AD groups, keeping quite stable as dementia progresses. This result might suggest that *ApoE* genotype effect is stronger before clinical manifestation of AD, being diluted when the disease appears. Previous EEG studies have assessed the importance of the left hemisphere in early stages of the dementia due to AD. It has been found that alpha coherence in AD patients decreases in temporal, parietal and occipital regions, being more pronounced in left areas [21]. Moreover, spectral analysis of the EEG showed that alterations in early stages of the disease might be more important in left temporal and parietal regions [69,70]. These results are particularly insteresting, since left hemisphere metabolic activity appears to be affected earlier than right one [51]. Our findings support the notion that left hemisphere showed accentuated alterations in early stages before AD development, supporting the important role of the *ApoE* risk allele in the AD neurodegenerative processes. In order to evaluate the genetic effect in these phases, several studies have analyzed the relation between different brain patterns and the presence of *ApoE* risk allele. Gorywala et al. [71] analyzed the small world properties in cognitively normal subjects. They observed that *ApoE*ε4 carriers showed altered connectivity patterns, compared to non-carriers. In addition, *ApoE*ε4 carriers reported a loss of hubs, seen primarily in the MTL, similar to other studies on MCI subjects. In another study, Dennis et al. [46] assessed the influence of the *ApoE*ε4 allele in the functional activation and the connectivity of the MTLs in young adults. They reported that *ApoE*ε4 carriers showed greater activation and reduced connectivity in the MTL than non-carriers, suggesting that ε4 carriers might require additional cognitive efforts than non-carriers in memory-related tasks to keep similar performance. Both studies hypothesized that the effect of the *ApoE* risk allele is more present before AD development, complementing our results from a different point of view.

### 4.4. Limitations and Future Research

Several issues have to be taken into consideration in future research. In this regard, a limitation may be the number of *ApoE* ε4 non-carriers (*n* = 151) when compared to carriers (*n* = 66). This may be due to the fact that ε3 allele is the most common allele (77%), and ε4 is only present at a frequency of ∼15% in the general population [72]. Furthermore, control carrier subgroup is formed by 6 subjects, whereas 10, 17, 17, and 16 patients have been analyzed in MCI, AD${}_{MIL}$, AD${}_{MOD}$, and AD${}_{SEV}$ carrier groups, respectively. This issue may be due to the higher percentage of *ApoE*ε4 alleles among AD patients (∼40%) [72]. In addition, we suggest that longitudinal studies of cognitively normal and MCI subjects could be included in future work, in order to check which patients progress to AD and to verify whether *ApoE* gene is involved in this process. Finally, since AD is considered a disconnection syndrome, we aim to conduct connectivity EEG analyses to evaluate the effects of *ApoE* genotypes in neural systems interconnection.

## 5. Conclusions

In this study, the influence of *ApoE* ε4 allele on the EEG complexity along the AD continuum was analyzed. Our results suggest that the complexity reduction associated with AD progression occurs in both ε4 carrier and non-carrier subgroups. However, EEG complexity patterns were statistically different between HC carriers and HC non-carriers, specially in left temporal region. This finding might be associated with a possible risk of dementia years before its clinical manifestation. These results highlight the relationship between EEG complexity and the presence of the *ApoE* risk allele in previous stages of dementia due to AD, which may be used to identify potential early AD biomarkers.

## Figures and Tables

**Table 1:** Socio-demographic and clinical data of the analyzed subjects, according to their *ApoE* ε4 dose. ${}^{a}$ Age (mean ± standard deviation); ${}^{b}$ MMSE score (mean ± standard deviation).

	*ApoE*ε4 Non-Carriers				*ApoE*ε4 Carriers			
Group	*N*	Age ${}^{a}$ (years)	Gender (Male:Female)	MMSE ${}^{b}$	*N*	Age ${}^{a}$ (years)	Gender (Male:Female)	MMSE ${}^{b}$
HC	40	79.32 ± 7.25	21:19	28.77 ± 1.12	6	83.33 ± 7.34	3:3	29.00 ± 1.26
MCI	39	85.41 ± 7.08	11:28	23.18 ± 3.11	10	83.70 ± 6.83	4:6	24.00 ± 1.76
AD${}_{MIL}$	28	79.86 ± 7.53	11:17	22.89 ± 2.45	17	80.41 ± 4.81	8:9	22.29 ± 2.02
AD${}_{MOD}$	27	82.26 ± 8.49	4:23	13.11 ± 2.99	17	79.29 ± 7.90	3:14	14.65 ± 2.55
AD${}_{SEV}$	17	80.59 ± 7.88	4:13	2.94 ± 3.78	16	81.00 ± 6.00	1:15	3.87 ± 4.22

## Abbreviations

The following abbreviations are used in this manuscript:

Aβ	Amyloid Beta protein
AD	Alzheimer’s Disease
AD${}_{MIL}$	Mild Alzheimer’s Disease
AD${}_{MOD}$	Moderate Alzheimer’s Disease
AD${}_{SEV}$	Severe Alzheimer’s Disease
ApoE	Apolipoprotein E
EEG	Electroencephalography
FDR	False Discovery Rate
FIR	Finite Impulse Response
fMRI	Functional Magnetic Resonance Imaging
ICA	Independent Component Analysis
LZC	Lempel-Ziv Complexity
MCI	Mild Cognitive Impairment
MMSE	Mini-Mental State Examination
MTL	Medial Temporal Lobe
NIA-AA	National Institute of Aging and Alzheimer’s Association
PET	Positron Emission Tomography
ROI	Region of Interest

## References

[1] Alzheimer’s Association (2019). 2019 Alzheimer ’s Disease Facts and Figures. Alzheimer’S Dement..

[2] Hebert L.E., Weuve J., Scherr P.A., Evans D.A. (2013). Alzheimer disease in the United States (2010-2050) estimated using the 2010 census. Neurology.

[3] Reisberg B., Ferris S., De Leon M., Crook T. (1982). The Global Deterioration Scale for Assessment of Primary Degenerative Dementia. Am. J. Psychiatry.

[4] Petersen R.C. (2016). Mild Cognitive Impairment. Continuum.

[5] Blennow K., Leon M.J.D., Zetterberg H. (2006). Alzheimer’s disease. Lancet.

[6] Jack C.R., Bennett D.A., Blennow K., Carrillo M.C., Dunn B., Haeberlein S.B., Holtzman D.M., Jagust W., Jessen F., Karlawish J. (2018). NIA-AA Research Framework: Toward a biological definition of Alzheimer’s disease. Alzheimer’S Dement..

[7] Zhu X.C., Tan L., Wang H.F., Jiang T., Cao L., Wang C., Wang J., Tan C.C., Meng X.F., Yu J.T. (2015). Rate of early onset Alzheimer’s disease: A systematic review and meta-analysis. Ann. Transl. Med..

[8] Jansen I.E., Savage J.E., Watanabe K., Bryois J., Williams D.M., Steinberg S., Sealock J., Karlsson I.K., Hägg S., Athanasiu L. (2019). Genome-wide meta-analysis identifies new loci and functional pathways influencing Alzheimer’s disease risk. Nat. Genet..

[9] Lambert J.C., Ibrahim-Verbaas C.A., Harold D., Naj A.C., Sims R., Bellenguez C., Jun G., DeStefano A.L., Bis J.C., Beecham G.W. (2013). Meta-analysis of 74,046 individuals identifies 11 new susceptibility loci for Alzheimer’s disease. Nat. Genet..

[10] Ridge P.G., Mukherjee S., Crane P.K., Kauwe J.S. (2013). Alzheimer’s disease: Analyzing the missing heritability. PLoS ONE.

[11] Bettens K., Sleegers K., Van Broeckhoven C. (2013). Genetic insights in Alzheimer’s disease. Lancet Neurol..

[12] Belloy M.E., Napolioni V., Greicius M.D. (2020). A Quarter Century of APOE and Alzheimer’s Disease: Progress to Date and the Path Forward. Neuron.

[13] Farrer L.A., Cupples L.A., Haines J.L., Hyman B., Kukull W.A., Mayeux R., Myers R.H., Pericak-Vance M.A., Risch N., Van Duijn C.M. (1997). Effects of age, sex, and ethnicity on the association between apolipoprotein E genotype and Alzheimer disease: A meta-analysis. J. Am. Med Assoc..

[14] Ewers M., Sperling R.A., Klunk W.E., Weiner M.W., Hampel H. (2011). Neuroimaging markers for the prediction and early diagnosis of Alzheimer’s disease dementia. Trends Neurosci..

[15] Babiloni C., Pizzella V., Gratta C.D., Ferretti A., Romani G.L. (2009). Chapter 5 Fundamentals of Electroencephalography. Magnetoencefalography, and Functional Magnetic Resonance Imaging.

[16] Sanei S., Chambers J.A. (2007). EEG Signal Processing.

[17] Phelps M.E. (2000). Positron emission tomography provides molecular imaging of biological processes. PNAS.

[18] Babiloni C., Cassetta E., Binetti G., Tombini M., Del Percio C., Ferreri F., Ferri R., Frisoni G., Lanuzza B., Nobili F. (2007). Resting EEG sources correlate with attentional span in mild cognitive impairment and Alzheimer’s disease. Eur. J. Neurosci..

[19] Babiloni C., Lizio R., Del Percio C., Marzano N., Soricelli A., Salvatore E., Ferri R., Cosentino F.I., Tedeschi G., Montella P. (2013). Cortical Sources of Resting State EEG Rhythms are Sensitive to the Progression of Early Stage Alzheimer’s Disease. J. Alzheimer’S Dis..

[20] Hampel H., Mesulam M.M., Cuello A.C., Khachaturian A.S., Farlow M.R., Snyder P.J., Giacobini E., Khachaturian Z.S. (2017). Revisiting the cholinergic hypothesis in Alzheimer’s disease: Emerging evidence from translational and clinical research. Alzheimer’S Dement..

[21] Locatelli T., Cursi M., Liberati D., Franceschi M., Comi G. (1998). EEG coherence in Alzheimer disease. Electroencephalogr. Clin. Neurophysiol..

[22] Abásolo D., Hornero R., Espino P., Poza J., Sánchez C.I., De La Rosa R. (2005). Analysis of regularity in the EEG background activity of Alzheimer’s disease patients with Approximate Entropy. Clin. Neurophysiol..

[23] Abásolo D., Hornero R., Espino P., Álvarez D., Poza J. (2006). Entropy analysis of the EEG background activity in Alzheimer’s disease patients. Physiol. Meas..

[24] Simons S., Espino P., Abásolo D. (2018). Fuzzy Entropy analysis of the electroencephalogram in patients with Alzheimer’s disease: Is the method superior to Sample Entropy?. Entropy.

[25] Maturana-Candelas A., Gómez C., Poza J., Pinto N., Hornero R. (2019). EEG characterization of the Alzheimer’s disease continuum by means of multiscale entropies. Entropy.

[26] Al-Nuaimi A.H.H., Jammeh E., Sun L., Ifeachor E. (2018). Complexity Measures for Quantifying Changes in Electroencephalogram in Alzheimer’s Disease. Complexity.

[27] Stam C.J., Jelles B., Achtereekte H.A., Van Birgelen J.H., Slaets J.P. (1996). Diagnostic Usefulness of Linear and Nonlinear Quantitative EEG Analysis in Alzheimer’s Disease. Clin. Eeg Neurosci..

[28] Jeong J., Kim S.Y., Han S.H. (1998). Non-linear dynamical analysis of the EEG in Alzheimer’s disease with optimal embedding dimension. Electroencephalogr. Clin. Neurophysiol..

[29] Cantero J.L., Atienza M., Cruz-Vadell A., Suarez-Gonzalez A., Gil-Neciga E. (2009). Increased synchronization and decreased neural complexity underlie thalamocortical oscillatory dynamics in mild cognitive impairment. NeuroImage.

[30] Ponomareva N.V., Korovaitseva G.I., Rogaev E.I. (2008). EEG alterations in non-demented individuals related to apolipoprotein E genotype and to risk of Alzheimer disease. Neurobiol. Aging.

[31] Canuet L., Tellado I., Couceiro V., Fraile C., Fernandez-Novoa L., Ishii R., Takeda M., Cacabelos R. (2012). Resting-State Network Disruption and APOE Genotype in Alzheimer’s Disease: A lagged Functional Connectivity Study. PLoS ONE.

[32] Kramer G., van der Flier W.M., de Langen C., Blankenstein M.A., Scheltens P., Stam C.J. (2008). EEG functional connectivity and ApoE genotype in Alzheimer’s disease and controls. Clin. Neurophysiol..

[33] Zappasodi F., Salustri C., Babiloni C., Cassetta E., Del Percio C., Ercolani M., Rossini P.M., Squitti R. (2008). An observational study on the influence of the APOE-*ϵ*4 allele on the correlation between ’free’ copper toxicosis and EEG activity in Alzheimer disease. Brain Res..

[34] Jeong J. (2004). EEG dynamics in patients with Alzheimer’s disease. Clin. Neurophysiol..

[35] Le Van Quyen M., Chavez M., Rudrauf D., Martinerie J. (2003). Exploring the nonlinear dynamics of the brain. J. Physiol. Paris.

[36] Costa M., Goldberger A.L., Peng C.K. (2005). Multiscale entropy analysis of biological signals. Phys. Rev..

[37] Abásolo D., Hornero R., Gómez C., García M., López M. (2006). Analysis of EEG background activity in Alzheimer’s disease patients with Lempel-Ziv complexity and central tendency measure. Med Eng. Phys..

[38] Gómez C., Hornero R., Abásolo D., Fernández A., López M. (2006). Complexity analysis of the magnetoencephalogram background activity in Alzheimer’s disease patients. Med Eng. Phys..

[39] Hornero R., Escudero J., Fernández A., Poza J., Gómez C. (2008). Spectral and nonlinear analyses of MEG background activity in patients with Alzheimer’s disease. IEEE Trans. Biomed. Eng..

[40] Gómez C., Hornero R., Abásolo D., Fernández A., Escudero J. (2009). Analysis of MEG background activity in Alzheimer’s disease using nonlinear methods and ANFIS. Ann. Biomed. Eng..

[41] Poza J., Gómez C., Bachiller A., Hornero R. (2012). Spectral and Non-Linear Analyses of Spontaneous Magnetoencephalographic Activity in Alzheimer’s Disease. J. Healthc. Eng..

[42] Fan J., Tao W., Li X., Li H., Zhang J., Wei D., Chen Y., Zhang Z. (2019). The contribution of genetic factors to cognitive impairment and dementia: Apolipoprotein E gene, gene interactions, and polygenic risk. Int. J. Mol. Sci..

[43] Shaw P., Lerch J.P., Pruessner J.C., Taylor K.N., Rose A.B., Greenstein D., Clasen L., Evans A., Rapoport J.L., Giedd J.N. (2007). Cortical morphology in children and adolescents with different apolipoprotein E gene polymorphisms: An observational study. Lancet Neurol..

[44] Bookheimer S.Y., Strojwas M.H., Cohen M.S., Saunders A.M., Pericak-Vance M.A., Mazziotta J.C., Small G.W. (2000). Patterns of brain activation in people at risk for Alzheimer’s disease. New Engl. J. Med..

[45] Borghesani P., Johnson L.C., Shelton A.L., Peskind E.R., Aylward E.H., Schelllenberg G.D., Cherrier M.M. (2008). Altered medial temporal lobe responses during visuospatial encoding in healthy APOE e4 carriers. Neurobiol. Aging.

[46] Dennis N.A., Browndyke J.N., Stokes J., Need A., James R., Welsh-bohmer K.A., Cabeza R. (2010). Temporal lobe functional activity and connectivity in young adult APOE e4 carriers. Alzheimer’S Dement..

[47] Machulda M.M., Jones D.T., Vemuri P., McDade E., Avula R., Przybelski S., Boeve B.F., Knopman D.S., Petersen R.C., Jack C.R. (2011). Effect of APOE *ϵ*4 Status on Intrinsic Network Connectivity in Cognitively Normal Elderly Subjects. Arch. Neurol..

[48] Duke Han S., Houston W., Jak A., Eyler L., Nagel B., Fleisher A., Brown G., Corey-Bloom J., Salmon D., Thal L. (2007). Verbal paired-associate learning by APOE genotype in non- demented older adults: fMRI evidence of a right hemispheric compensatory response. Neurobiol. Aging.

[49] Squire L.R., Stark C.E., Clark R.E. (2004). The Medial Temporal Lobe. Annu. Rev. Neurosci..

[50] Braak H., Braak E. (1997). Diagnostic criteria for neuropathologic assessment of Alzheimer’s disease. Neurobiol. Aging.

[51] Loewenstein D.A., Barker W.W., Chang J.Y., Apicella A., Yoshii F., Kothari P., Levin B., Duara R. (1989). Predominant left hemisphere metabolic dysfunction in dementia. Arch. Neurol..

[52] Albert M.S., DeKosky S.T., Dickson D., Dubois B., Feldman H.H., Fox N.C., Gamst A., Holtzman D.M., Jagust W.J., Petersen R.C. (2011). The Diagnosis of Mild Cognitive Impairment due to Alzheimer’s Disease: Recommendations from the National Institute on Aging-Alzheimer’s Association Workgroups on Diagnostic Guidelines for Alzheimer’s Disease. Alzheimer’S Dement..

[53] McKhann G.M., Knopman D.S., Chertkow H., Hyman B.T., Jack C.R., Kawas C.H., Klunk W.E., Koroshetz W.J., Manly J.J., Mayeux R. (2011). The diagnosis of dementia due to Alzheimer’s disease: Recommendations from the National Institute on Aging- Alzheimer’s Association workgroups on diagnostic guidelines for Alzheimer’s disease. Alzheimer’S Dement..

[54] Folstein M.F., Folstein S.E., McHugh P.R. (1975). “Mini-mental state”. J. Psychiatr. Res..

[55] Ruiz-Gómez S.J., Gómez C., Poza J., Martínez-Zarzuela M., Tola-Arribas M.A., Cano M., Hornero R. (2018). Measuring alterations of spontaneous EEG neural coupling in alzheimer’s disease and mild cognitive impairment by means of cross-entropy metrics. Front. Neuroinformatics.

[56] Lempel A., Ziv J. (1976). On the Complexity of Finite Sequences. IEEE Trans. Inf. Theory.

[57] Zhang X.S., Roy R.J., Jensen E.W. (2001). EEG complexity as a measure of depth of anesthesia for patients. IEEE Trans. Biomed. Eng..

[58] Nagarajan R. (2002). Quantifying physiological data with Lempel-Ziv complexity-Certain issues. IEEE Trans. Biomed. Eng..

[59] Benjamini Y., Hochberg Y. (1995). Controlling the False Discovery Rate: A Practical and Powerful Approach to Multiple Testing. J. R. Stat. Soc. Ser. (Methodological).

[60] Dozolme D., Prigent E., Yang Y.F., Amorim M.A. (2018). The neuroelectric dynamics of the emotional anticipation of other people’s pain. PLoS ONE.

[61] Luft F., Sharifi S., Mugge W., Schouten A.C., Bour L.J., Van Rootselaar A.F., Veltink P.H., Heida T. (2020). Distinct cortical activity patterns in Parkinson’s disease and essential tremor during a bimanual tapping task. J. Neuroeng. Rehabil..

[62] Dauwels J., Srinivasan K., Ramasubba Reddy M., Musha T., Vialatte F.B., Latchoumane C., Jeong J., Cichocki A. (2011). Slowing and Loss of Complexity in Alzheimer’s EEG: Two Sides of the Same Coin?. Int. J. Alzheimer’S Dis..

[63] Zhu B., Chai C., Gao S., Ren H., Cao L., Dong Z., Geng X., Zheng J., Qian X., Bi X. (2017). Analysis of EEG Complexity in Patients with Mild Cognitive Impairment. J. Neurol. Disord..

[64] Gaubert S., Raimondo F., Houot M., Corsi M.C., Naccache L., Diego Sitt J., Hermann B., Oudiette D., Gagliardi G., Habert M.O. (2019). EEG evidence of compensatory mechanisms in preclinical Alzheimer’s disease. Brain.

[65] Nakamura A., Cuesta P., Fernández A., Arahata Y., Iwata K., Kuratsubo I., Bundo M., Hattori H., Sakurai T., Fukuda K. (2018). Electromagnetic signatures of the preclinical and prodromal stages of Alzheimer’s disease. Brain.

[66] McBride J.C., Zhao X., Munro N.B., Smith C.D., Jicha G.A., Hively L., Broster L.S., Schmitt F.A., Kryscio R.J., Jiang Y. (2014). Spectral and complexity analysis of scalp EEG characteristics for mild cognitive impairment and early Alzheimer’s disease. Comput. Methods Programs Biomed..

[67] Escudero J., Abásolo D., Hornero R., Espino P., Lopez M. (2006). Analysis of electroencephalograms in Alzheimer’s disease patients with multiscale entropy. Physiol. Meas..

[68] Lipsitz L.A., Goldberger A.L. (1992). Loss of ‘Complexity’ and Aging. J. Am. Med Assoc..

[69] Dierks T., Perisic I., Frölich L., Ihl R., Maurer K. (1991). Topography of the quantitative electroencephalogram in dementia of the Alzheimer type: Relation to severity of dementia. Psychiatry Res. Neuroimaging.

[70] Smailovic U., Koenig T., Kåreholt I., Andersson T., Kramberger M.G., Winblad B., Jelic V. (2018). Quantitative EEG power and synchronization correlate with Alzheimer’s disease CSF biomarkers. Neurobiol. Aging.

[71] Goryawala M., Duara R., Loewenstein D.A., Zhou Q., Barker W., Adjouadi M., The Alzheimer’s Disease Neuro (2015). Apolipoprotein-E4 (ApoE4) carriers show altered small-world properties in the default mode network of the brain. Biomed. Phys. Eng. Express.

[72] Bu G. (2009). Apolipoprotein e and its receptors in Alzheimer’s disease: Pathways, pathogenesis and therapy. Nat. Rev. Neurosci..

